# Effects of opioid-free and opioid-sparing anesthesia on postoperative delirium: A systematic review and meta-analysis

**DOI:** 10.1097/MD.0000000000051469

**Published:** 2026-11-06

**Authors:** Tingting Liu, Xiu Geng, Lijuan Wang, Ruirui Sang, Haizhen Huang

**Affiliations:** a Department of Anesthesiology, Central Hospital Affiliated to Shandong First Medical University, Jinan, Shandong Province, China.

**Keywords:** dexmedetomidine, esketamine, ketamine, opioid-free anesthesia, postoperative delirium, systematic review and meta-analysis

## Abstract

**Background:**

Postoperative delirium (POD) is a common neurocognitive condition linked with increased morbidity, long-term cognitive decline, and death. Perioperative opioid exposure may contribute to delirium through sedation, neuroinflammation, and neurotransmitter dysregulation. Opioid-free anesthesia and opioid-sparing anesthesia using agents such as ketamine, esketamine, and dexmedetomidine have emerged as a potential strategy to reduce POD. This meta-analysis evaluated the effects of opioid-free and opioid-sparing anesthetic regimens on POD in adult surgical patients.

**Methods:**

Embase, PubMed, and Web of Science were searched from inception to April 2026. Randomized controlled trials comparing ketamine-, esketamine-, or dexmedetomidine-based opioid-free and opioid-sparing strategies with placebo, conventional opioid-based anesthesia, or standard care were included. The primary outcome was POD. Risk ratios (RRs) with 95% confidence intervals (CIs) were pooled using a random-effects model.

**Results:**

Forty-one randomized controlled trials involving 7852 patients were analyzed. POD occurred in 488 of 4013 patients receiving opioid-free/opioid-sparing interventions and 633 of 3839 controls. Overall, these strategies significantly reduced the risk of delirium (RR = 0.641, 95% CI: 0.525–0.784; *I*^2^ = 52.2%). Subgroup analyses showed reductions with esketamine (RR = 0.745, 95% CI: 0.526–1.053) and dexmedetomidine (RR = 0.612, 95% CI: 0.471–0.796), while ketamine demonstrated a nonsignificant trend toward benefit (RR = 0.598, 95% CI: 0.251–1.423). No significant publication bias was detected.

**Conclusions:**

Opioid-free and opioid-sparing anesthetic strategies reduce POD in adult surgical patients, with dexmedetomidine and esketamine showing the most consistent benefits. These findings support multimodal opioid-sparing anesthesia for perioperative neurocognitive protection.

**Key Points**

- Question: Do opioid-free and opioid-sparing anesthetic strategies reduce the risk of postoperative delirium in adult surgical patients compared with conventional opioid-based anesthesia?
- Findings: In this systematic review and meta-analysis of 41 randomized controlled trials involving 7852 patients, opioid-free and opioid-sparing anesthetic strategies significantly reduced the risk of postoperative delirium, with the most consistent benefits observed for dexmedetomidine- and esketamine-based regimens.
- Meaning: These findings support the incorporation of multimodal opioid-sparing anesthetic strategies, particularly dexmedetomidine- and esketamine-based protocols, to reduce postoperative delirium and improve perioperative neurocognitive outcomes in adult surgical patients.

## 1. Introduction

Postoperative delirium (POD) remains a major perioperative neurocognitive complication and is frequently encountered in surgical patients, particularly among older adults and those with critical illness.^[1]^ The condition is characterized by rapidly developing and fluctuating disturbances in attention, awareness, and cognition that commonly occur within days of an operation. Incidence rates vary according to patient characteristics and procedural factors but may exceed 50% in high-risk groups, including older adults undergoing major cardiac, orthopedic, or abdominal surgery.^[2]^ Beyond its immediate clinical manifestations, POD is associated with numerous adverse consequences, such as longer durations of mechanical ventilation, prolonged hospitalization, greater healthcare expenditure, long-term neurocognitive impairment, poorer quality of life, and increased mortality.^[3]^ As a result, prevention of POD has become a key focus of perioperative management strategies.

The pathophysiology of POD is multifactorial and incompletely understood.^[4]^ Proposed mechanisms include neuroinflammation, neurotransmitter imbalance, oxidative stress, sleep disruption, cerebral hypoperfusion, and perioperative pain.^[5,6]^ Exposure to opioids during the perioperative period has also been implicated as a potential contributor to delirium development through mechanisms involving excessive sedation, respiratory depression, altered neurotransmission, and disruption of central cholinergic pathways. Opioid administration may additionally exacerbate postoperative sleep disturbances and cognitive dysfunction, particularly in older adults who possess reduced neurophysiological reserve. These concerns have led to growing interest in anesthetic strategies that minimize or eliminate perioperative opioid exposure.^[7,8]^

Opioid-free anesthesia (OFA) has gained increasing attention as a promising perioperative approach aimed at avoiding intraoperative opioid administration while maintaining adequate analgesia and hemodynamic stability through the use of multimodal nonopioid agents.^[9]^ OFA protocols commonly incorporate agents including dexmedetomidine, magnesium sulfate, ketamine, regional anesthetic techniques, lidocaine, and nonsteroidal anti-inflammatory drugs. Among these agents, dexmedetomidine and ketamine have received considerable attention because of their potential neuroprotective and opioid-sparing properties. Dexmedetomidine, a selective α2-adrenergic agonist, possesses sedative, analgesic, anxiolytic, and anti-inflammatory effects while preserving respiratory drive.^[10,11]^ Several studies have suggested that dexmedetomidine may reduce delirium incidence through improved sleep architecture, attenuation of neuroinflammation, and reduced opioid consumption.^[12–14]^ Similarly, low-dose ketamine and esketamine may provide analgesia while reducing central sensitization and opioid requirements.^[15]^ Experimental evidence also suggests that ketamine may modulate neuroinflammatory pathways implicated in delirium pathogenesis.

Despite increasing adoption of OFA techniques, evidence regarding their effectiveness in reducing POD remains inconsistent. Some trials have shown reductions in POD with dexmedetomidine- or ketamine-based opioid-sparing protocols, whereas others have reported neutral or conflicting findings.^[12–14,16,17]^ Furthermore, variations in surgical populations, anesthetic protocols, delirium assessment tools, dosing regimens, and follow-up periods have contributed to heterogeneity across studies. Previous systematic reviews have often focused on individual agents such as dexmedetomidine or ketamine alone, specific surgical populations, or outcomes other than delirium.^[18]^ Therefore, a comprehensive synthesis evaluating the overall effects of opioid-free and opioid-sparing anesthetic strategies on POD across adult surgical patients remains necessary.

Given the increasing clinical emphasis on enhanced recovery pathways, opioid-free and opioid-sparing perioperative care, clarification of the relationship between OFA and POD is of substantial clinical importance. Identifying anesthetic strategies capable of reducing delirium may improve perioperative outcomes, particularly in vulnerable elderly and high-risk surgical populations. To address this knowledge gap, we conducted a systematic review and meta-analysis to assess the effects of OFA, including ketamine-, esketamine-, and dexmedetomidine-based regimens, on the incidence of POD in adult surgical patients undergoing various surgical procedures.

## 2. Methods

### 2.1. Study design and reporting guidelines

Reporting of this review followed the Preferred Reporting Items for Systematic Reviews and Meta-Analyses framework.^[19]^ The study aimed to evaluate the effects of OFA and opioid-sparing anesthesia, including ketamine-, esketamine-, and dexmedetomidine-based anesthetic regimens, on the incidence of POD in adult surgical patients. The review protocol was not prospectively registered.

### 2.2. Ethical considerations

As this study was a systematic review and meta-analysis based exclusively on data from previously published studies, no new patient recruitment or collection of individual patient data was undertaken. Therefore, ethical approval from an institutional review board or ethics committee and informed consent were not required.

### 2.3. Search strategy

A systematic search of PubMed, Embase, and Web of Science was conducted from database inception through May 2026. Search terms included controlled vocabulary (MeSH and Emtree terms) together with relevant free-text keywords related to POD, OFA, ketamine, esketamine, dexmedetomidine, and adult surgical populations. The search strategy was tailored to the indexing system and search functionality of each database. To ensure comprehensive study identification, the reference lists of relevant reviews and eligible articles were also examined manually for additional citations. Detailed search strategies are provided in Table S1, Supplemental Digital Content 1.

### 2.4. Eligibility criteria

Studies were included if they met the following criteria: randomized controlled trials (RCTs) involving adult surgical patients aged 18 years or older; studies comparing opioid-free or opioid-sparing anesthetic regimens containing ketamine, esketamine, or dexmedetomidine with conventional opioid-based anesthesia, placebo, or standard perioperative care; studies reporting POD as an outcome; and studies published in English. Studies involving pediatric populations, observational studies, case reports, conference abstracts, editorials, reviews, animal studies, and nonsurgical patients were excluded. Studies focusing exclusively on postoperative cognitive dysfunction without formal delirium assessment were excluded from quantitative synthesis.

### 2.5. Study selection

Study selection was undertaken independently by 2 investigators. Following title and abstract screening, potentially eligible studies were subjected to full-text review based on predefined inclusion and exclusion criteria. Differences in reviewer judgments were settled through consensus. When multiple publications reported data from the same patient cohort, only the report providing the most comprehensive information was included in the meta-analysis.

### 2.6. Data extraction

Two reviewers independently extracted study data using a standardized collection form. Extracted variables comprised study authorship, publication year, country of origin, trial design, characteristics of the surgical cohort, operative procedure, intervention and control regimens, participant numbers, delirium assessment methodology, follow-up period, and reported delirium outcomes.

POD incidence was designated as the primary outcome. Diagnosis and assessment of delirium were based on validated screening and diagnostic tools, such as Confusion Assessment Method (CAM), Confusion Assessment Method for the Intensive Care Unit (CAM-ICU), Intensive Care Delirium Screening Checklist (ICDSC), Delirium Observation Screening Scale, and other established instruments used to evaluate postoperative neurocognitive status.

### 2.7. Risk of bias assessment

Methodological rigor was assessed using the risk of bias 2 (ROB-2) instrument recommended by the Cochrane Collaboration. The evaluation examined potential bias related to randomization, adherence to assigned interventions, missing data, outcome assessment, and reporting practices. Each trial received an overall judgment of low risk, some concerns, or high risk of bias. Two investigators independently completed the assessments, and disagreements were settled through consensus.

### 2.8. Statistical analysis

Data synthesis was performed in R software (R Foundation, Vienna, Austria). Dichotomous outcomes were expressed as risk ratios (RRs) with accompanying 95% confidence intervals (CIs). Heterogeneity across studies was examined using the Cochran *Q* statistic and the *I*^2^ metric, with *I*^2^ values >50% considered indicative of meaningful inconsistency.

Because clinical and methodological differences were anticipated across the included studies, a random-effects framework was applied for all pooled analyses. Additional subgroup analyses were conducted according to intervention type. The robustness of the findings was evaluated through sensitivity analyses that excluded studies with an elevated risk of bias or zero-event outcomes when applicable. Funnel plot assessment was used to investigate potential publication bias when sufficient studies were available. All statistical tests were two-sided, and *P* < .05 was considered statistically significant.

## 3. Results

### 3.1. Literature search

A total of 2657 citations were retrieved from the electronic databases, including PubMed (n = 867), Embase (n = 1005), and Web of Science (n = 785). After deduplication, 1307 unique records underwent title and abstract review. Screening led to the exclusion of 1230 records that were not relevant to the research question.

A total of 77 articles were evaluated in full text for inclusion, with no studies excluded because of retrieval issues. Following full-text review, 36 articles were excluded for the following reasons: wrong outcome reporting (n = 22), inappropriate study design (n = 2), evaluation of unrelated drugs or interventions (n = 11), and retracted study (n = 1). Ultimately, 41 RCTs^[12–14,16,17,20–55]^ satisfied the inclusion criteria and contributed to the qualitative and quantitative evidence synthesis (Fig. 1).

**Figure 1.**
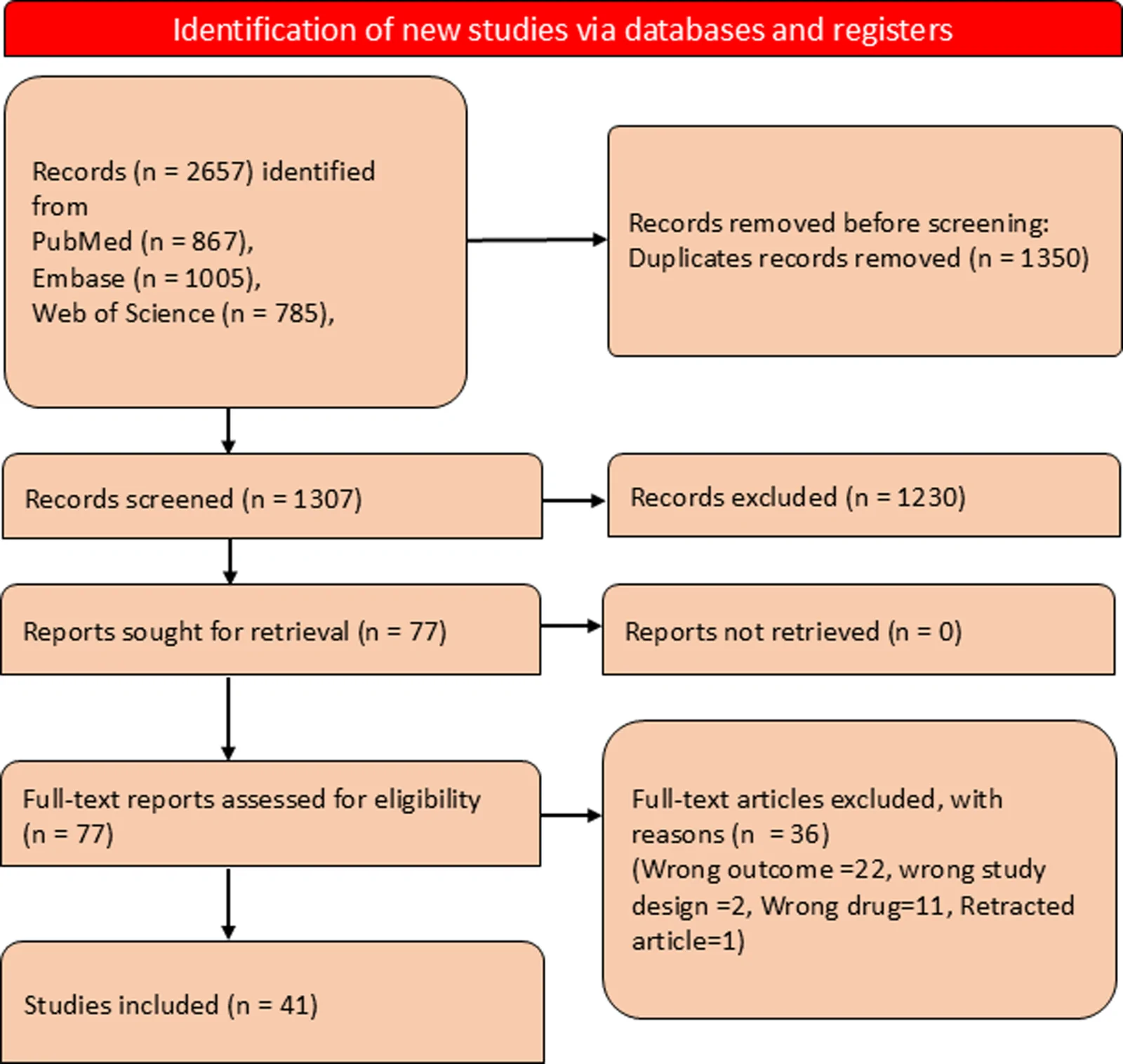
Preferred Reporting Items for Systematic Reviews and Meta-Analyses flow diagram depicting article selection and screening process.

### 3.2. Characteristics of included studies

A total of 41 RCTs^[12–14,16,17,20–55]^ involving adult surgical patients were considered in the current evidence synthesis. Publication years of the included studies ranged from 2003 to 2025 and evaluated the effects of ketamine-, esketamine-, or dexmedetomidine-based opioid-free or opioid-sparing anesthetic strategies on POD and perioperative neurocognitive outcomes (Table 1).

**Table 1:** Characteristics of included studies.

Author	Year	Drug	Surgery type	Comparator	POD tool
Avidan MS^[20]^	2017	Ketamine	Major surgery	Placebo	CAM
Farhadi K^[21]^	2025	Ketamine	Colorectal surgery	Control	CAM-ICU
Gecaj-Gashi A^[22]^	2010	Ketamine	General surgery	Placebo	Unclear
Ghazaly HF^[23]^	2023	Ketamine	Emergency surgery	Placebo	DOS
Hollinger A^[16]^	2021	Ketamine	Mixed surgery	Placebo	DOS/Nu-DESC/ICDSC
Hudetz JA^[24]^	2009	Ketamine	Cardiac surgery	Placebo	ICDSC
Kornilov E^[25]^	2024	Ketamine	DBS surgery	Propofol	Unclear
Lee KH^[26]^	2015	Ketamine	Orthopedic surgery	Control	MMSE
Oriby ME^[27]^	2023	Ketamine	Cataract surgery	Dexmedetomidine/control	Cognitive tests
Pereira H^[28]^	2023	Ketamine	Laparoscopic surgery	Placebo	Nu-DESC
Urban MK^[29]^	2008	Ketamine	Spinal fusion	Control	CAM
Huang C^[30]^	2024	Esketamine	Noncardiac cancer surgery	Placebo	CAM
Ju LY^[31]^	2025	Esketamine	CABG	Placebo	CAM-ICU/3D-CAM
Li J^[32]^	2022	Esketamine	Elderly surgery	Control	CAM
Luo T^[33]^	2024	Esketamine	Thoracic surgery	Placebo	CAM/MMSE
Ma CB^[34]^	2024	Esketamine	Hip/knee arthroplasty	Placebo	D-CAM
Wang H^[14]^	2024	Esketamine	Mastectomy surgery	Placebo	Unclear
Xiong X^[35]^	2024	Esketamine	Cardiac valve surgery	Placebo	CAM/CAM-ICU
Zhou D^[36]^	2023	Esketamine	Breast/thyroid surgery	Placebo	Unclear
Zhang Y^[17]^	2024	Esketamine	Elective noncardiac surgery	Placebo	3D-CAM
Azeem T^[37]^	2018	Dexmedetomidine	Cardiac surgery	Morphine + Midazolam	CAM-ICU
Chitnis S^[38]^	2021	Dexmedetomidine	CABG/aortic valve surgery	Propofol	ICDSC
Djaiani G^[39]^	2016	Dexmedetomidine	Coronary/valve surgery	Propofol	CAM/CAM-ICU
Dong CH^[40]^	2021	Dexmedetomidine	Elective cardiac surgery	Placebo	CAM-ICU
Herr D^[41]^	2003	Dexmedetomidine	CABG	Propofol	Not specified
Ji F^[42]^	2014	Dexmedetomidine	CABG	Control	Delirium outcome
Likhvantsev V^[43]^	2021	Dexmedetomidine	CABG/valve surgery	Placebo	CAM-ICU/ICDSC
Maldonado J^[44]^	2009	Dexmedetomidine	Valve surgery	Propofol/Midazolam	CAM/CAM-ICU
Momeni M^[45]^	2021	Dexmedetomidine	On-pump cardiac surgery	Placebo	CAM-ICU
Park J^[46]^	2014	Dexmedetomidine	Open cardiac surgery	Remifentanil	CAM-ICU
Preveden M^[47]^	2023	Dexmedetomidine	On-pump cardiac surgery	Propofol	CAM-ICU
Priye S^[48]^	2015	Dexmedetomidine	Cardiac surgery	Placebo	RASS
Qu J^[49]^	2023	Dexmedetomidine	Elective cardiac surgery	Placebo	CAM/CAM-S
Shehabi Y ^[50]^	2009	Dexmedetomidine	On-pump cardiac surgery	Morphine	CAM-ICU/MAAS
Subramaniam B^[51]^	2019	Dexmedetomidine	CABG ± valve surgery	Propofol	CAM/CAM-ICU
Susheela A^[12]^	2017	Dexmedetomidine	CABG/valve surgery	Propofol	MMSE/CAM
Turan A^[52]^	2020	Dexmedetomidine	On-pump cardiac surgery	Placebo	CAM-ICU
van Norden J^[13]^	2021	Dexmedetomidine	CABG	Placebo	CAM-ICU/ICDSC
Wang H^[14]^	2023	Dexmedetomidine	Heart valve surgery	Placebo	CAM-ICU
Eremenko A^[53]^	2014	Dexmedetomidine	Cardiac surgery	Placebo	CAM-ICU
Gao Y^[55]^	2020	Dexmedetomidine	Off-pump CABG	Placebo	CAM-ICU
Imran-ul-Hassan S^[54]^	2021	Dexmedetomidine	Elective cardiac surgery	Placebo	CAM-ICU

3D-CAM = 3-Minute Diagnostic Interview for Confusion Assessment Method, CABG = coronary artery bypass grafting, CAM = Confusion Assessment Method, CAM-ICU = Confusion Assessment Method for the Intensive Care Unit, CAM-S = Confusion Assessment Method–Severity, DBS = deep brain stimulation, D-CAM = Diagnostic Confusion Assessment Method, DOS = Delirium Observation Screening Scale, ICDSC = Intensive Care Delirium Screening Checklist, MAAS = Motor Activity Assessment Scale, MMSE = Mini-Mental State Examination, Nu-DESC = Nursing Delirium Screening Scale, POD = postoperative delirium, RASS = Richmond Agitation-Sedation Scale.

A large proportion of the included studies involved patients undergoing cardiac procedures, especially coronary artery bypass grafting (CABG), valvular operations, and other surgeries performed with cardiopulmonary bypass support. Noncardiac populations encompassed a broad range of procedures, including orthopedic, thoracic, laparoscopic, colorectal, cataract, breast, and thyroid surgery, spinal fusion, deep brain stimulation surgery, and major abdominal or cancer-related procedures.

Ketamine-based studies primarily examined low-dose intraoperative ketamine use aimed at reducing the risk of POD and cognitive impairment. These studies included elderly patients undergoing major procedures, orthopedic procedures, spinal fusion, emergency surgery, laparoscopic surgery, and cardiac surgery. Most ketamine studies compared ketamine with placebo or standard perioperative care, while one study compared ketamine with dexmedetomidine and another evaluated propofol-ketamine anesthesia during deep brain stimulation surgery. Assessment of delirium and cognitive function was performed using established and validated measures, including CAM, ICDSC, Delirium Observation Screening Scale, Mini-Mental State Examination, Nursing Delirium Screening Scale, and other recognized neurocognitive evaluation tools.

Esketamine studies were more recent and were predominantly published between 2022 and 2025. These studies evaluated perioperative esketamine administration in older surgical patients treated with thoracic surgery, hip or knee arthroplasty, noncardiac cancer surgery, cardiac valve surgery, off-pump CABG, mastectomy, and elective noncardiac surgery. Most esketamine studies compared the intervention with placebo or standard anesthesia protocols. Delirium was commonly assessed using CAM, CAM-ICU, 3-Minute Diagnostic Interview for Confusion Assessment Method, D-CAM, Mini-Mental State Examination, or combined neurocognitive assessment strategies.

Dexmedetomidine represented the most extensively studied intervention in the present review. The majority of dexmedetomidine trials involved patients receiving cardiac surgical interventions, including CABG and heart valve procedures. Comparator regimens included placebo, propofol, morphine-based sedation, remifentanil, midazolam, or conventional postoperative care. Several studies evaluated postoperative intensive care unit sedation with dexmedetomidine, whereas others investigated perioperative or nocturnal dexmedetomidine infusion protocols. Delirium assessment was most frequently performed using CAM-ICU, CAM, ICDSC, CAM-S, or related validated screening instruments.

The included studies demonstrated considerable heterogeneity with respect to patient populations, anesthetic protocols, dosing regimens, timing of intervention administration, delirium assessment methods, and duration of follow-up. Nevertheless, most studies focused on elderly or high-risk surgical populations who were considered particularly vulnerable to postoperative neurocognitive complications. Study enrollment differed markedly across trials, with sample sizes ranging from small exploratory studies involving fewer than 20 participants to large multicenter randomized trials enrolling several hundred patients.

Overall, the included evidence reflects growing clinical interest in opioid-free and opioid-sparing anesthetic strategies for prevention of POD and optimization of perioperative neurocognitive outcomes in surgical patients.

### 3.3. Quality of studies

Study quality was evaluated using the ROB-2 tool. Most trials were judged to have a low risk of bias or only minor methodological concerns, indicating generally satisfactory study quality. Several large multicenter RCTs, including studies by Avidan MS, Hollinger A, Huang C, Djaiani G, Dong CH, Qu J, and Turan A, demonstrated a low risk of bias across each ROB-2 assessment domain. These studies employed appropriate randomization procedures, adequate blinding strategies, complete outcome reporting, and validated delirium assessment tools. Some trials raised minor concerns owing to inadequate descriptions of randomization concealment and blinding procedures, or potential selective outcome reporting. Studies including Hudetz JA, Urban MK, Lee KH, Pereira H, Chitnis S, Herr D, Park J, and Priye S fell into this category. In these trials, concerns were mainly related to deviations from intended interventions, subjective outcome assessment, or incomplete clarification of prespecified analyses. Only one study (Maldonado et al) was considered at high risk of bias because of concerns regarding randomization and outcome assessment. Most trials reported complete follow-up data, resulting in a low risk of attrition bias. Similarly, measurement of outcomes was considered reliable in most studies because validated delirium screening instruments such as CAM, CAM-ICU, ICDSC, and related neurocognitive assessment tools were frequently utilized. Overall, the included evidence demonstrated moderate-to-high methodological quality, with most studies showing a low risk of bias or only minor methodological concerns.

### 3.4. Meta-analysis

A total of 41 RCTs involving 7852 participants were included in the pooled quantitative analysis evaluating the effects of opioid-free or opioid-sparing anesthetic strategies on POD. Across all included studies, POD occurred in 488 of 4013 patients in the intervention groups and 633 of 3839 patients in the control groups (Fig. 2).

**Figure 2.**
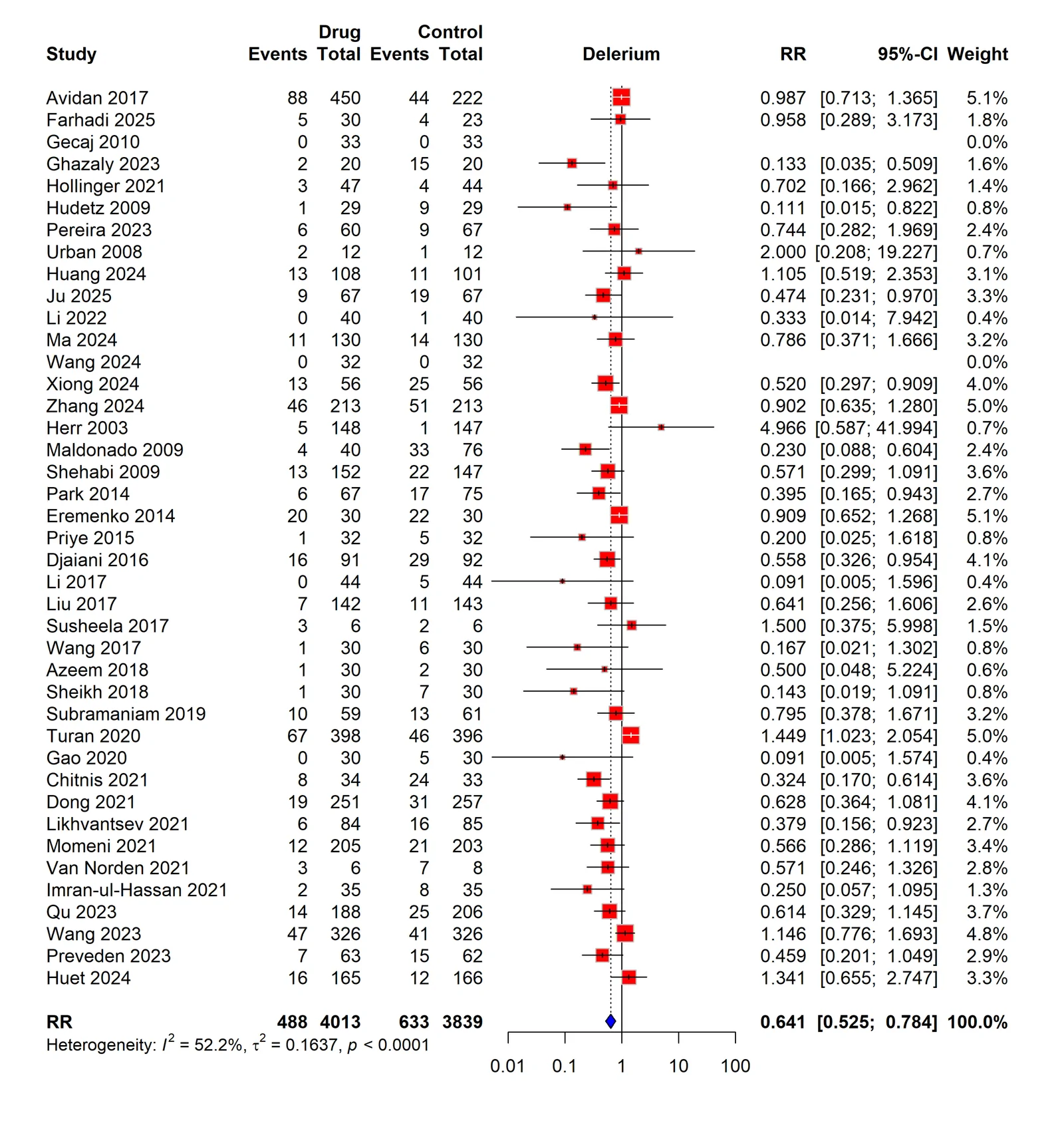
Forest plot demonstrating the overall effect of opioid-free and opioid-sparing anesthetic strategies on postoperative delirium in adult surgical patients. CI = confidence interval, RR = risk ratio.

The random-effects meta-analysis demonstrated that opioid-free or opioid-sparing anesthetic strategies based on ketamine, esketamine, or dexmedetomidine were associated with a statistically significant reduction in POD compared with control interventions. The overall pooled RR was 0.641 (95% CI: 0.525–0.784), corresponding to an approximately 36% relative reduction in the risk of POD. Moderate statistical heterogeneity was observed across the included studies (*I*^2^ = 52.2%, τ^2^ = 0.1637, *P* < .0001).

### 3.5. Subgroup analysis

Subgroup analyses were conducted according to the pharmacological agent used in the opioid-free or opioid-sparing anesthetic regimen, including ketamine, esketamine, and dexmedetomidine (Fig. 3).

**Figure 3.**
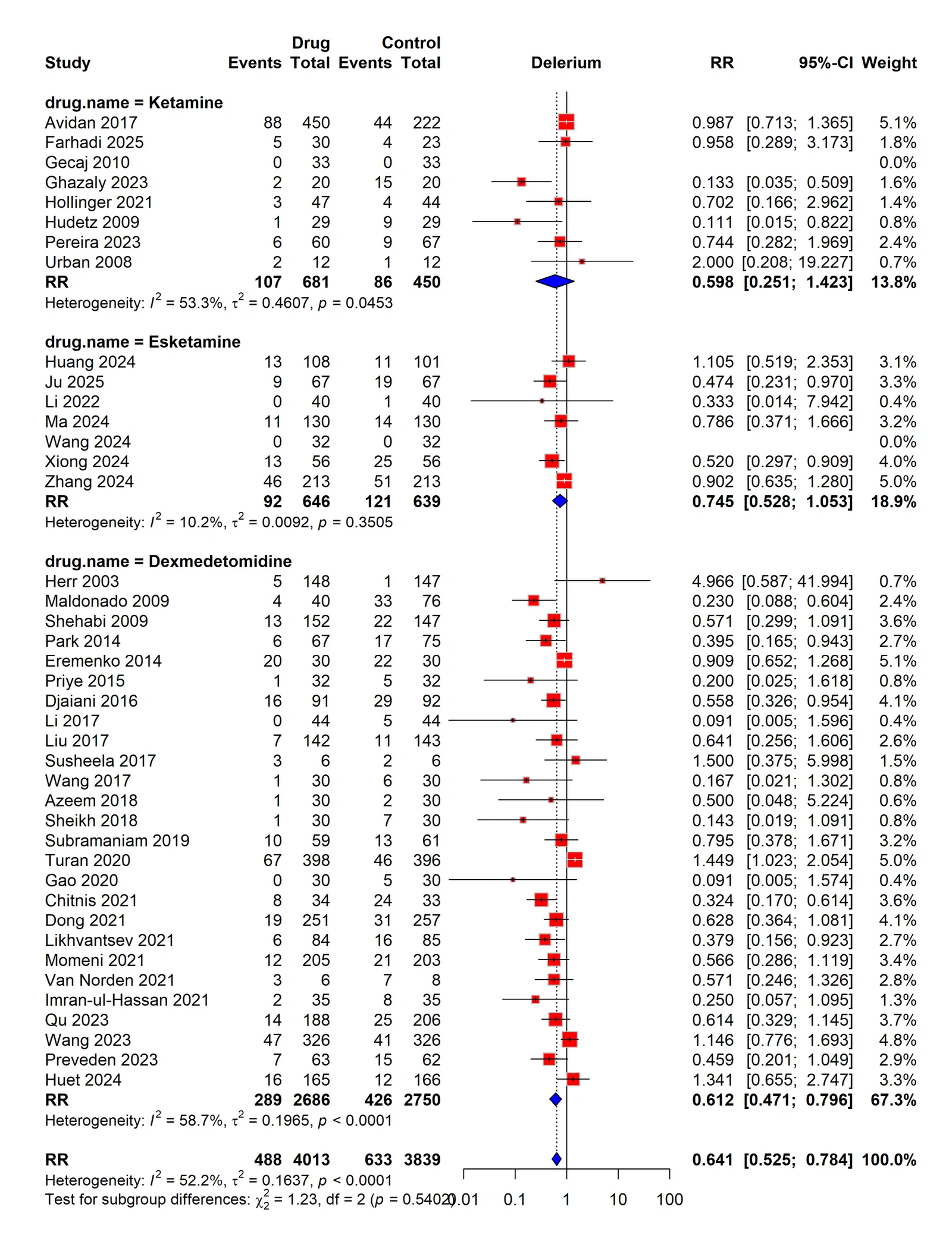
Subgroup analysis forest plot evaluating the effects of ketamine-, esketamine-, and dexmedetomidine-based opioid-free anesthetic strategies on postoperative delirium in adult surgical patients. CI = confidence interval, RR = risk ratio.

The ketamine subgroup comprised 8 RCTs involving 1131 patients, of whom 681 received ketamine and 450 received control interventions. POD occurred in 107 patients in the ketamine group and 86 patients in the control group. The pooled estimate suggested a potential reduction in POD with ketamine-based anesthesia; however, the difference was not statistically significant (RR = 0.598, 95% CI: 0.251–1.423). Moderate heterogeneity was observed across the included studies (*I*^2^ = 53.3%, *P* = .0453).

The esketamine subgroup included 7 RCTs involving 1285 patients, with 646 patients receiving esketamine and 639 receiving control interventions. POD occurred in 92 patients in the esketamine group and 121 patients in the control group. Esketamine-based anesthetic strategies showed a trend toward reduced POD, although the pooled effect did not reach statistical significance (RR = 0.745, 95% CI: 0.528–1.053). Heterogeneity was low across the included studies (*I*^2^ = 10.2%, *P* = .3505), suggesting relatively consistent effect estimates.

The dexmedetomidine subgroup comprised 26 RCTs involving 5436 patients, including 2686 patients in the dexmedetomidine group and 2750 in the control group. POD occurred in 289 patients receiving dexmedetomidine and 426 patients receiving control interventions. The pooled analysis demonstrated a statistically significant reduction in POD with dexmedetomidine-based regimens (RR = 0.612, 95% CI: 0.471–0.796), corresponding to an approximately 39% relative reduction in risk. Moderate-to-substantial heterogeneity was observed across the included studies (*I*^2^ = 58.7%, *P* < .0001).

### 3.6. Publication bias

Potential publication bias was evaluated through visual examination of funnel plots and quantitatively using Egger’s regression analysis. The funnel plot appeared largely symmetrical around the overall effect estimate, with no obvious evidence of asymmetry that would indicate meaningful small-study effects (Fig. 4).

**Figure 4.**
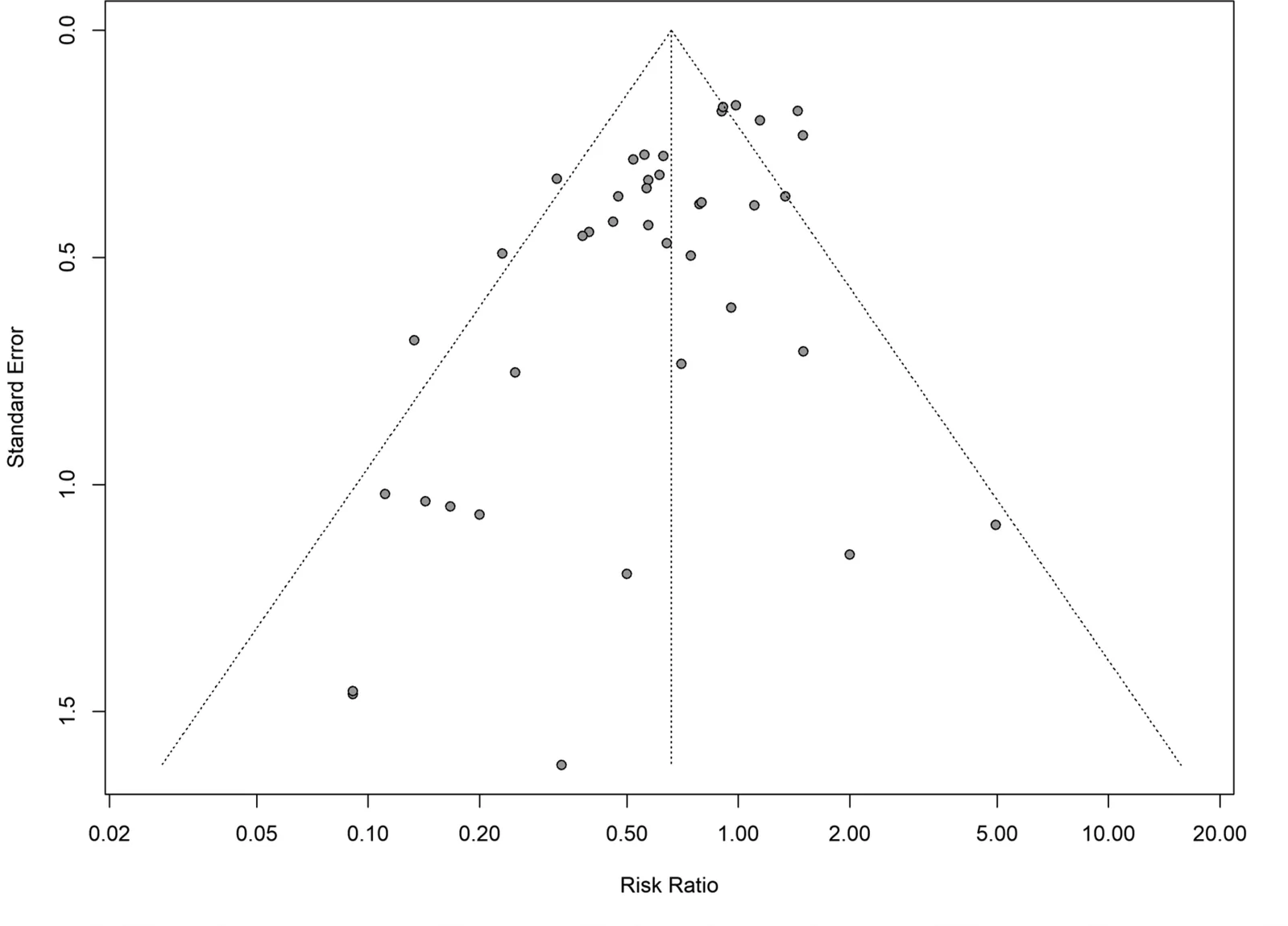
Funnel plot assessing publication bias among included studies evaluating ketamine-, esketamine-, and dexmedetomidine-based opioid-free anesthetic strategies and postoperative delirium.

Assessment using Egger’s regression method failed to detect significant publication bias (*P* > .05). Accordingly, there was no clear evidence that reporting bias or small-study effects materially influenced the overall findings of the meta-analysis.

## 4. Discussion

A total of 41 RCTs involving 7852 patients were included in the meta-analysis. The overall findings demonstrated that opioid-free and opioid-sparing anesthetic regimens based on ketamine, esketamine, and dexmedetomidine were associated with a significant reduction in POD compared with conventional anesthetic approaches. Overall, patients receiving opioid-free or opioid-sparing anesthetic strategies experienced an approximately 36% relative reduction in the risk of POD. These findings suggest that minimizing perioperative opioid exposure through multimodal anesthetic approaches may offer potential neurocognitive benefits, particularly among older adults and patients at high risk of POD.

POD remains a major perioperative complication connected with increased morbidity, longer durations of hospitalization, subsequent cognitive deterioration, institutionalization, and death.^[56]^ Although the pathogenesis of delirium is multifactorial, perioperative opioid exposure has increasingly been implicated as a modifiable contributor. Opioids may precipitate delirium through several mechanisms, including excessive sedation, respiratory depression, sleep disruption, cholinergic dysfunction, and neuroinflammatory activation.^[57]^ Consequently, opioid-sparing anesthesia has emerged as a promising strategy within enhanced recovery protocols aimed at reducing opioid-related adverse effects while maintaining adequate analgesia and hemodynamic stability.

The findings derived from the pooled analysis are consistent with the growing body of literature supporting opioid-sparing and opioid-free anesthetic strategies for mitigating POD. Previous systematic reviews have demonstrated that dexmedetomidine significantly reduces the risk of delirium in surgical and intensive care populations through its sedative, analgesic, sympatholytic, and anti-inflammatory properties.^[58,59]^ Similarly, recent RCTs evaluating esketamine have reported favorable effects on postoperative neurocognitive outcomes, particularly in older adults undergoing major cardiac and noncardiac surgical procedures.^[60,61]^ Our findings extend these observations by providing a comprehensive evaluation of multiple opioid-free agents across diverse surgical populations and demonstrating an overall 36% reduction in POD risk. In contrast, evidence regarding ketamine has remained inconsistent. While some studies reported significant reductions in delirium following low-dose ketamine administration, particularly in cardiac surgical patients,^[24,62]^ larger trials such as the PODCAST study failed to demonstrate a protective effect.^[20]^ The nonsignificant benefit observed in our ketamine subgroup likely reflects heterogeneity in patient populations, dosing strategies, and outcome assessment methods. Importantly, unlike previous reviews that focused on individual agents or specific surgical settings, the present study synthesizes evidence across ketamine-, esketamine-, and dexmedetomidine-based regimens, thereby providing broader support for multimodal opioid-sparing anesthesia as an effective strategy for perioperative neurocognitive protection and delirium prevention.^[63]^

The evidence synthesized in the present meta-analysis supports growing evidence that opioid-sparing anesthetic techniques may improve postoperative neurocognitive outcomes. Among the evaluated agents, dexmedetomidine demonstrated the strongest and most consistent protective effect against POD. The dexmedetomidine subgroup exhibited a statistically meaningful reduction in delirium rates, with findings driven largely by studies involving cardiac surgical populations. Dexmedetomidine possesses several pharmacologic properties that may explain these beneficial effects. As a selective α2-adrenergic agonist, it provides sedation resembling physiologic sleep, reduces sympathetic activation, attenuates systemic inflammation, and decreases perioperative opioid requirements. Experimental and clinical studies have additionally suggested that dexmedetomidine may preserve sleep architecture, reduce neuronal apoptosis, and modulate neuroinflammatory pathways implicated in delirium pathogenesis.

Importantly, many dexmedetomidine studies included in the present review involved elderly individuals undergoing bypass-assisted cardiac surgery, a population recognized to be at particularly high risk for POD because of advanced age, systemic inflammation, microembolic events, and prolonged intensive care unit exposure. The observed benefits within this subgroup, therefore, carry substantial clinical relevance. Nevertheless, moderate-to-high heterogeneity remained present among dexmedetomidine studies, likely reflecting variations in infusion timing, dosing protocols, comparator regimens, delirium assessment methods, and perioperative care pathways.

Esketamine-based strategies also demonstrated a statistically significant reduction in POD with relatively low heterogeneity across studies. As the pharmacologically active S-isomer of ketamine, esketamine exhibits greater binding affinity for N-methyl-D-aspartate receptors and may achieve analgesic and anti-inflammatory effects at lower doses than the racemic formulation. In addition to reducing opioid consumption, esketamine may attenuate central sensitization and neuroinflammation while maintaining cardiovascular stability. The relatively consistent findings among esketamine studies are notable and may reflect increasing standardization of dosing regimens and delirium assessment tools in recent trials. As most esketamine studies were published within the last few years, these findings suggest an emerging role for esketamine within multimodal opioid-sparing anesthesia protocols.

In contrast, ketamine-based interventions demonstrated a trend toward reduced POD, although statistical significance was not achieved in subgroup analysis. Several factors may explain these findings. First, ketamine studies demonstrated substantial heterogeneity with respect to surgical populations, dosing regimens, timing of administration, and outcome assessment. Second, some included ketamine trials were based on relatively small study populations, thereby reducing analytical power. Third, ketamine possesses psychotomimetic properties that may theoretically contribute to postoperative neuropsychiatric symptoms in certain patients, particularly at higher doses. Nevertheless, several important studies included in the analysis, particularly among cardiac surgical populations, demonstrated clinically meaningful reductions in delirium risk with low-dose ketamine administration. Accordingly, the lack of statistical significance should not necessarily be considered indicative of inefficacy.

An important consideration in interpreting these findings is the clinical heterogeneity across the included trials. Differences in patient characteristics, type and complexity of surgery, perioperative risk profiles, intervention dose and timing, comparator anesthetic regimens, and methods used to diagnose POD may have contributed to the observed variability in treatment effects. This heterogeneity is particularly relevant when considering the generalizability of the findings to routine clinical practice. A substantial proportion of the included studies, especially those evaluating dexmedetomidine, involved patients undergoing cardiac surgery, who may have a different baseline risk of POD and perioperative care pathway compared with patients undergoing noncardiac procedures. Therefore, the observed benefits, particularly for dexmedetomidine, should be interpreted cautiously when extrapolating to other surgical populations. Differences in delirium assessment tools and timing of outcome assessment may also influence the reported incidence and treatment effects. Future trials should use standardized delirium definitions and assessment methods and should evaluate diverse surgical populations to improve the applicability of these findings across different clinical settings.

The present study possesses several important strengths. First, this meta-analysis collated a large number of RCTs and more than 7000 surgical patients, thereby providing substantial statistical power. Second, the study evaluated multiple opioid-free anesthetic agents across diverse surgical populations, allowing comprehensive assessment of opioid-sparing perioperative strategies. Third, only RCTs were included, enhancing the overall quality of evidence. In addition, evaluation of study quality indicated that most included trials were methodologically robust, while publication bias assessment did not identify significant small-study effects. However, a few limitations should also be acknowledged. Some studies were limited by modest sample sizes, single-center designs, and restricted durations of follow-up. The inability to perform detailed dose-response analyses or evaluate interactions between multiple nonopioid agents represents an additional limitation.

The evidence synthesized in this study has important implications for contemporary perioperative clinical care. Opioid-free and opioid-sparing anesthetic strategies may be considered as components of a multimodal approach to POD prevention, particularly in older adults and patients at increased risk of neurocognitive complications. By reducing perioperative opioid exposure while maintaining adequate analgesia and anesthesia, these approaches may provide an additional strategy for minimizing modifiable perioperative risk factors for delirium. However, they should be implemented alongside other established perioperative measures for delirium prevention rather than being considered as a standalone intervention.

Among the evaluated agents, dexmedetomidine and esketamine were associated with statistically significant reductions in POD, suggesting potential clinical utility within appropriately selected perioperative protocols. The findings for dexmedetomidine are supported by a relatively larger evidence base, although moderate-to-high heterogeneity indicates that its effect may vary according to surgical population, dosing regimen, timing of administration, and comparator strategy. Esketamine also demonstrated a significant reduction in delirium with relatively low heterogeneity; however, the evidence is more recent and comparatively limited. In contrast, ketamine demonstrated a favorable but nonsignificant effect, and therefore the current evidence does not support recommending ketamine specifically for delirium prevention on the basis of the present findings alone.

In clinical practice, the choice of an opioid-sparing strategy should therefore be individualized according to patient characteristics, baseline delirium risk, type and extent of surgery, anticipated analgesic requirements, hemodynamic status, and potential adverse effects. Appropriate opioid-sparing approaches may also be incorporated into enhanced recovery after surgery pathways when clinically suitable. Importantly, these strategies should complement comprehensive perioperative care, including appropriate pain management, avoidance of unnecessary sedation, optimization of physiological factors, and routine assessment for delirium in high-risk patients. Further large, well-designed RCTs are warranted to determine the optimal agent, dose, timing, and patient population for opioid-free and opioid-sparing strategies and to establish their long-term clinical and economic benefits.

Future large RCTs are needed to determine the most effective dosing strategies, timing of administration, and patient selection criteria for opioid-free anesthetic strategies. Additional research should also evaluate long-term neurocognitive outcomes, functional recovery, cost-effectiveness, and the comparative effectiveness of different opioid-sparing protocols across various surgical populations.

## 5. Conclusion

Opioid-free and opioid-sparing anesthetic strategies were found to significantly lower the risk of POD among adult surgical patients. Dexmedetomidine and esketamine demonstrated the most consistent protective effects, whereas ketamine showed a favorable but nonsignificant trend. These findings support the growing role of multimodal opioid-sparing anesthesia in perioperative neurocognitive protection and POD prevention.

## Author contributions

**Conceptualization:** Tingting Liu, Haizhen Huang.

**Data curation:** Tingting Liu, Xiu Geng.

**Methodology:** Tingting Liu, Lijuan Wang.

**Formal analysis:** Tingting Liu, Lijuan Wang.

**Writing – original draft:** Tingting Liu.

**Writing – review & editing:** Xiu Geng, Ruirui Sang, Haizhen Huang.


