# Supplementary material for: Effects of opioid-free and opioid-sparing anesthesia on postoperative delirium: A systematic review and meta-analysis
Source: Medicine (Baltimore). 2026 Nov 6;105(45):e51469. doi: 10.1097/MD.0000000000051469 (PMC13747511; doi:10.1097/MD.0000000000051469)
Supplement: Supplementary file 1 [file medi-105-e51469-s001.docx]

**Table S1: Search strategy**

| Database | Search Strategy |
| --- | --- |
| **PubMed** | ("Postoperative Delirium"[Mesh] OR "Delirium"[Mesh] OR postoperative delirium OR post-operative delirium OR POD OR emergence delirium OR postoperative cognitive dysfunction OR POCD OR perioperative neurocognitive disorder OR POND) AND ("Ketamine"[Mesh] OR ketamine OR esketamine OR s-ketamine OR "Dexmedetomidine"[Mesh] OR dexmedetomidine) AND ("Surgical Procedures, Operative"[Mesh] OR surgery OR surgical OR perioperative OR postoperative OR intraoperative OR anesthesia OR anaesthesia) AND (randomized controlled trial[pt] OR controlled clinical trial[pt] OR randomized[tiab] OR randomised[tiab] OR placebo[tiab] OR trial[tiab] OR groups[tiab])) NOT ((animals[mh]) NOT (humans[mh])) |
| **Embase** | ('postoperative delirium'/exp OR 'delirium'/exp OR 'postoperative delirium' OR 'post operative delirium' OR pod OR 'emergence delirium' OR 'postoperative cognitive dysfunction' OR pocd OR 'perioperative neurocognitive disorder' OR pond) AND ('ketamine'/exp OR ketamine OR esketamine OR 's-ketamine' OR 'dexmedetomidine'/exp OR dexmedetomidine) AND ('surgery'/exp OR 'surgical procedure'/exp OR surgery OR surgical OR perioperative OR postoperative OR intraoperative OR anesthesia OR anaesthesia) AND ('randomized controlled trial'/exp OR 'clinical trial'/exp OR random*:ti,ab OR placebo:ti,ab OR trial:ti,ab)) NOT ('animal'/exp NOT 'human'/exp) |
| **Web of Science** | TS=(("postoperative delirium" OR "post-operative delirium" OR POD OR "emergence delirium" OR "postoperative cognitive dysfunction" OR POCD OR "perioperative neurocognitive disorder" OR POND OR delirium) AND (ketamine OR esketamine OR "s-ketamine" OR dexmedetomidine) AND (surgery OR surgical OR perioperative OR postoperative OR intraoperative OR anesthesia OR anaesthesia) AND (randomized OR randomised OR placebo OR "clinical trial" OR trial)) |
